# Integrative Bioinformatics Analysis Revealed Mitochondrial Dysfunction-Related Genes Underlying Intervertebral Disc Degeneration

**DOI:** 10.1155/2022/1372483

**Published:** 2022-10-11

**Authors:** Zhengya Zhu, Zhongyuan He, Tao Tang, Fuan Wang, Hongkun Chen, Baoliang Li, Guoliang Chen, Jianmin Wang, Wei Tian, Dafu Chen, Xinbao Wu, Xizhe Liu, Zhiyu Zhou, Shaoyu Liu

**Affiliations:** ^1^Innovation Platform of Regeneration and Repair of Spinal Cord and Nerve Injury, Department of Orthopaedic Surgery, The Seventh Affiliated Hospital, Sun Yat-Sen University, Shenzhen 518107, China; ^2^Guangdong Provincial Key Laboratory of Orthopedics and Traumatology, Orthopaedic Research Institute/Department of Spinal Surgery, The First Affiliated Hospital of sun Yat-Sen University, Guangzhou 510080, China; ^3^Laboratory of Bone Tissue Engineering, Beijing Laboratory of Biomedical Materials, Beijing Research Institute of Orthopaedics and Traumatology, Beijing Jishuitan Hospital, Beijing 100035, China

## Abstract

**Objective:**

Mitochondrial dysfunction plays an important role in intervertebral disc degeneration (IDD). We aim to explore the pathways and key genes that cause mitochondrial dysfunction during IDD and to further reveal the pathogenesis of IDD based on bioinformatic analyses.

**Methods:**

Datasets GSE70362 and GSE124272 were downloaded from the Gene Expression Omnibus. Differentially expressed genes (DEGs) of mitochondrial dysfunction between IDD patients and healthy controls were screened by package limma package. Critical genes were identified by adopting gene ontology (GO), Kyoto encyclopedia of genes and genomes (KEGG) pathways, and protein-protein interaction (PPI) networks. We collected both degenerated and normal disc tissues obtained surgically, and we performed western blot and qPCR to verify the key DEGs identified in intervertebral disc tissues.

**Results:**

In total, 40 cases of IDD and 24 healthy controls were included. We identified 152 DEGs, including 67 upregulated genes and 85 downregulated genes. Four genes related to mitochondrial dysfunction (SOX9, FLVCR1, NR5A1 and UCHL1) were screened out. Of them, SOX9, FLVCR1, and UCHL1 were down-regulated in peripheral blood and intervertebral disc tissues of IDD patients, while NR5A1 was up-regulated. The analysis of immune infiltration showed the concentrations of mast cells activated were significantly the highest in IDD patients. Compared with the control group, the level of T cells CD4 memory resting was the lowest in the patients. In addition, 24 cases of IDD tissues and 12 cases of normal disc tissues were obtained to verify the results of bioinformatics analysis. Both western blot and qPCR results were consistent with the results of bioinformatics analysis.

**Conclusion:**

We identified four genes (SOX9, FLVCR1, NR5A1 and UCHL1) associated with mitochondrial dysfunction that play an important role in the progress of disc degeneration. The identification of these differential genes may provide new insights for the diagnosis and treatment of IDD.

## 1. Introduction

Low back pain (LBP) is a common muscle bone disease that causes physical pains to patients and severe economic burden to the society [1, 2]. Intervertebral disc degeneration (IDD), as one major cause of LBP, is featured by structural destruction, apoptosis of nucleus pulposus cells, release of proinflammatory cytokines, and extracellular matrix degradation in the intervertebral disc [3, 4]. So far, the concrete pathology of IDD is unclear, and the clinical treatment strategies are mainly targeted at conservative treatment or surgical intervention for symptom relieving. Nevertheless, nearly 20% of IDD patients respond badly to non-surgical therapy [5]. Thus, further clarifying the molecular mechanism of IDD is a new route for precise intervention of LBP.

A normal intervertebral disc is anatomically composed of gelatinous nucleus pulposus (NP) in the center and annulus fibrosus tissues in the periphery, and is assisted by cartilaginous endplates (CEP) to enhance its mechanical strength. The intervertebral disc (especially nucleus pulposus tissues) is critical in maintaining the physiological functions of the spine, and can absorb and disperse the machinery loads of the spine that moves at all directions [6]. Intervertebral disc, the largest vessel-free organ in the human body, acquires energy mainly through anaerobic glycolysis [7], and mitochondria are critical in supply energy for the intervertebral disc to maintain normal physiological functions [5]. In addition to matter and energy metabolism, mitochondria are involved in regulation and control of second messenger functions, such as release of reactive oxygen species (ROS) and calcium ions, and further activate various signaling pathways to maintain the steady functions of nucleus pulposus cells. Reportedly, the mitochondria in the NP cells of IDD patients are structurally and functionally abnormal, and mitochondrial dysfunction may be one of the causes that accelerate the progression of IDD. As a by-product of aerobic respiration, ROS is mainly produced in the mitochondria by the electron transport chain and other mitochondrial located proteins. With the excessive accumulation of ROS, mitochondria are the main target of ROS attack in disc cells [8].

In this study, we screened out the differential expressions of mitochondrial genes, between normal people and IDD patients in both intervertebral disc tissues and peripheral blood, from the transcriptome sequencing gene data of Gene Expression Omnibus (GEO). The key pathways and proteins were identified from analysis of gene ontologies (GOs), Kyoto Encyclopedia of Genes and Genomes (KEGG) pathways, protein-protein interaction (PPI) networks, and some other bioinformatics analysis tools that offer a new clue to uncover the pathogenesis of IDD and theoretically underlies intervertebral disc repair and regeneration.

## 2. Materials and Methods

### 2.1. Data Downloading

Datasets GSE70362 [9] and GSE124272 [10, 11] were downloaded from GEO. GSE70362 containing 32 cases of IDD and 16 control cases was obtained from the GPL17810 sequencing platform. The GSE124272 containing 8 cases of IDD and 8 control cases was acquired from the GPL21185 sequencing platform. All cases were of human source. The above two datasets were integrated for downstream analysis. The batch effect between datasets was calibrated using package SVA of R language [12] and the data were normalized using log2. The expression distribution after the above processing was visualized in box plots, including 40 IDD cases and 24 control cases.

To analyze the expressions of mitochondrial dysfunction genes in all samples, we first identified 11 mitochondrial dysfunction genes from database GeneCards [13] by using keyword ‘Mitochondrial dysfunction' (CGB5, KIT, FLVCR1, SP1, SOX9, UCHL1, CYP21A2, NR5A1, DAZ4, DHH, POMK). Then after intersection with the existing expression profiles, 9 genes were left (KIT, FLVCR1, SP1, SOX9, UCHL1, CYP21A2, NR5A1, DHH, POMK).

### 2.2. Panorama of Mitochondrial Dysfunction Genes

To further explore the correlations of mitochondrial dysfunction genes in all patients, we calculated the Pearson correlations between genes. The absolute value of correlation coefficient larger than 0.3 and p <0.05 indicate the presence of correlation. The correlations between qualified genes were plotted on the R package ggplot2 [14].

To analyze the effects of expressions of mitochondrial functional genes on IDD, we analyzed the differentially expressed genes (DEGs) between the IDD group and the control group using the integrated dataset on the R package limma [15] and screened out the significant genes. The thresholds were absolute value of log2 (fold change) (log2FC) >1.5 and Padj<0.05. The genes with log2FC>1.5 and Padj<0.05 were upregulated, and the genes with log2FC< -1.5 and Padj<0.05 were downregulated. Especially, the volcano plots show the downregulated or upregulated DEGs. The heatmaps of all these DEGs in all samples were plotted on the R package pheatmap [16]. To analyze the expressions of mitochondrial dysfunction genes between the control and tested groups, we plotted the box plots of the two groups using the R package ggpubr [17]. The two groups were compared using Wilcoxon rank sum test. The significant level was p <0.05.

Protein-protein interaction (PPI) networks are formed from the interactions of single proteins, and participate in all steps of the life process, including biosignal transfer, gene expression regulation, energy and substance metabolism, and cell cycle regulation. Systematic analysis of interactions among abundant proteins in biosystems is contributive to understanding the rationale of proteins in biosystems, clarifying the mechanisms of biosignals and energy/substance metabolism in special physiological states such as diseases, and for knowing the functional associations between proteins. STRING is a database for searching known PPIs and predicting PPIs [18]. We used STRING and chose the genes with combined score larger than 400 to build a network of interactions between DEG-related proteins. The PPI network was visualized on Cytoscape 3.7.2 [19].

### 2.3. Diagnostic Model Based on Mitochondrial Dysfunction Genes

Given the influence of mitochondrial dysfunction, the control samples and tested samples may contain different mitochondrial dysfunction genes that show different statuses. Hence, it is highly feasible to build a diagnostic model based on mitochondrial dysfunction genes.

Here, we first used ridge regression to screen all mitochondrial dysfunction genes on the R package glmnet [20], and found out the optimal *λ*. After the regression, the genes with coefficient not being 0 were remained. Then the genes were further screened through logistics regression. The genes chosen for modeling and their coefficients were displayed as forest map on the R package forestplot [21].

After that, to identify the multifactor influence of feature genes in the diagnostic model, we chose the genes with large absolute weights in the previous model on the R package rms [22] and built a new logistics multifactor regression model. To validate the predictive efficacy of the new diagnostic model, we plotted receiver's operating characteristic curve (ROC) and calculated the area under curve (AUC) on the R package pROC [23].

### 2.4. Gene Set Enrichment Analysis

To further uncover the biological differences between the tested samples and the control samples, we subjected the DEGs to gene set enrichment analysis (GSEA).

GSEA is a commonly-used method for large-scale function enrichment analysis at different dimensions and levels, and usually involves three aspects: bioprocesses, molecular functions, and cell components [24]. KEGG is a widely-used database that stores information of genome, biological pathways, diseases, and drugs [25]. All significant DEGs were subjected to GO function annotation and KEGG pathway enrichment analyses on the R package clusterprofiler [26, 27] to identify the significantly enriched bioprocesses. The results were visualized as bar charts and bubble plots. The significant threshold of enrichment analysis was set at adjusted p <0.05.

Gene enrichment analysis determines whether a preset gene is significantly different between two biological states, and is often used to estimate the changes in pathways and bioprocesses in a dataset [28]. To study the differences in bioprocesses between two groups, we chose the gene profile dataset, and downloaded reference gene sets ‘c5.go.v7.4.entrez.gmt' and ‘c2.cp.kegg.v7.4. entrez.gmt' from database MSigDB [29]. The datasets were enriched and visualized using the GSEA from the R package clusterprofiler. The significant level was set at adjusted p <0.05.

GSVA, the gene set variation analysis [30] and a nonparametric unsupervised method, converts the between-sample gene expression matrix into a between-sample gene set expression matrix, and thereby evaluates the gene set enrichment of chip nucleolus transcriptome. GSVA is used to evaluate whether a pathway is enriched between samples. Gene sets ‘c5.go.v7.4.entrez.gmt' and ‘c2.cp.kegg.v7.4. entrez.gmt' acquired from database MSigDB were sent to GSVA at the gene expression level, and thereby, the function enrichment differences were compared between two types of tissues.

### 2.5. WGCNA

Weighted gene correlation network analysis (WGCNA), a systematic biological method to describe gene association modes between samples, can identify the gene sets with highly collaborative changes, and identify the candidate biological marker genes or therapeutic targets according to the internality of gene sets and to the associations between gene sets and phenotypes [31]. The correlated key gene sets between the tested and control groups were identified using the R package WGCNA [31], which were used in subsequent analysis.

### 2.6. Protein-Protein Interaction (PPI) Network

The gene expressions are universally and mutually associated, and especially, the genes that regulate the same biological process are highly associated. Hence, to uncover the gene associations in the tested group or the control group through WGCNA, we built a PPI network.

The above genes from the database STRING [32] were inputted to build a PPI network at the default confidence level of 0.4. Then the PPI was outputted. Further analysis was done on Cytoscape [19]. The network attributes of nodes were calculated, and the hub nodes were identified with node degrees as the standard on the package Cytohubba [33]. The top 10 nodes ranked by the node degree were classified as the hub nodes. These nodes were highly associated with other nodes and thus may play critical roles in the regulation and control of all biological processes, which are worth of further research.

### 2.7. Identification and Correlation Analysis of Immune Infiltrating Cells

The immune microenvironment mainly consists of immune cells, inflammatory cells, fibroblasts, interstitium samples, and various cytokines and chemokines, and thus is a loaded comprehensive system. The infiltration analysis of immune cells is pivotal in disease research and prediction of therapeutic prognosis. CIBERSORT is an algorithm for deconvolution of expression matrix of immune cell subtypes according to the rationale of linear support vectors, and uses RNA-Seq data to estimate the abundance of immune cells in samples [34]. The CIBERSORT from R language [34] was used to calculate the abundance of 22 types of immune cells between the tested group and the control group, and the composition of immune cells was visualized as box plots. The differences in proportions of immune cells were calculated using Wilcoxon test, at the significant level of P <0.05.

### 2.8. Unsupervised Clustering

Because of ubiquitous heterogeneity among patients, such heterogeneity can be interpreted using unsupervised clustering of samples according to hub genes, and the samples were reclassified. This classification can help us to comprehensively understand the mechanism of mitochondrial dysfunction in different modes of disc degeneration.

First, the optimal number of clusters was determined using the R package factoextra [35]. After that, all patients were clustered in an unsupervised way using k-mean clustering. The samples were classified into 2 clusters, and the final clustering effect was examined using factoextra. The expressions of the 10 hub genes between the two groups were displayed as heatmaps. Histograms of groups were plotted on R package ggpubr [17] with the sample clustering label. The two groups were compared using Wilcoxon rank sum test at the significant level p <0.05.

### 2.9. RNA Extraction and Real-Time Quantitative Polymerase Chain Reaction (RT-qPCR)

The ethics approvals were provided by the institutional review board of the Seventh Affiliated Hospital of Sun Yat-sen University (KY-2021-030-01). All enrolled patients provided written informed consent for the research protocol. The degree of IDD was determined by magnetic resonance imaging (MRI) following Pfirrmann classification [36]. Tissues of Pfirrmann I-II were used as controls. Human lumbar disc tissues were obtained from patients who underwent spinal canal decompression treatment. The nucleus pulposus and annulus fibrosus tissues were separated and RNA was extracted directly from tissues according to a previous protocol [37]. In brief, 150 mg of a sample was cut up and then digested with 2 mg/ml pronase at 37°C, flash-frozen, pulverized in liquid nitrogen, and homogenized with a tissue lyser. Total RNA was extracted using a TRI Reagent (Invitrogen, USA) and 400 ng of RNA was then converted to cDNA using a cDNA synthesis kit (Takara, Japan).

RT-qPCR was performed using Power Up SYBR Green Master Mix (Thermo Fisher Scientific, USA) on a real-time system (Bio-Rad, USA). As described in the protocol, each reaction mixture consisted of 5 *μ*l of 2 × Power Up SYBR Green Master Mix, 2 *μ*l of nuclease-free water, 0.5 *μ*l of each of 10 *μ*M forward and reverse primers and 2 *μ*l of cDNA. The applied cycle conditions were: 50°C for 2 min and 95°C for 2 min followed by 44 cycles of 15 s at 95°C and 1 min at 60°C. The specific primers used here were designed using Primer 6.0 (Applied Biosystems, CA), and the sequences are provided in Table 1. Results were normalized to housekeeping gene glyceraldehyde 3-phosphate dehydrogenase (GAPDH) expression using the 2^-*ΔΔ*Ct^ algorithm.

### 2.10. Extraction and Culture of Primary Intervertebral Disc Cells

NP tissues and AF tissues were digested using 2 mg/ml type II collagenase (Gibco, USA) at 37°C. After washing with PBS, the digested tissues were transferred to DMEM/F12 (Gibco, USA) containing 10% fetal bovine serum (Gibco, USA) and 1% penicillin/streptomycin (Gibco, USA) in the incubator at 5% CO_2_ and 37°C. The cells at the confluent stage were passaged after digestion with 0.25% Trypsin-EDTA (Gibco, USA). Cells after the second passage were used in the following experiments.

### 2.11. Total Protein Extraction and Western Blot

Total proteins were extracted with an RIPA buffer (Thermo Fisher Scientific, USA) and the concentrations were determined by a BCA kit (Boster, China). Proteins were electrophoresed in premade polyacrylamide gels containing SDS, and transferred to polyvinylidene fluoride(PVDF)membranes from the gels. After blocking with 5% non-fat milk (Solarbio, China) for 1 h, the PVDF membranes were incubated with primary antibody overnight at 4°C and then incubated with secondary antibody (coupled with horseradish peroxidase) for 1 h at room temperature. The protein signal was visualized by an ECL chemiluminescence kit (EpiZyme, China), and the grayscale of band was quantified using Image J.

### 2.12. Statistical Analysis

All data were analyzed and processed on R 4.1.1. Continuous variables in normal distribution were compared between groups using independent Student t test, and those in nonnormal distribution were compared using Mann–Whitney *U* test (namely Wilcoxon rank sum test). Variables between groups were statistically analyzed using Chi-square test or Fisher's exact test. The correlation coefficients between genes were calculated via Pearson correlation analysis. All *P* values are two-sided. P <0.05 indicates significance.

## 3. Results

### 3.1. Data Processing

**Figure 1:**
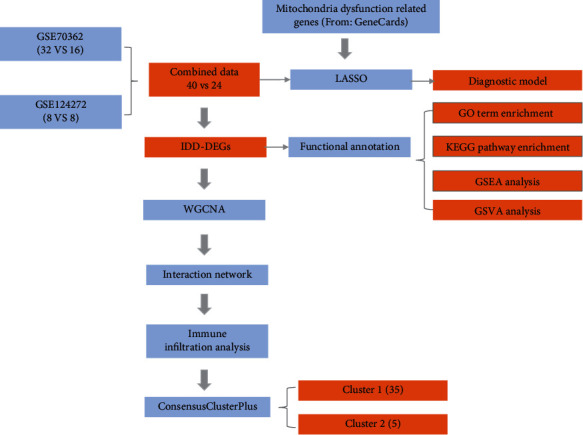
Flow diagram presenting the main plan and process of the study.

**Figure 2:**
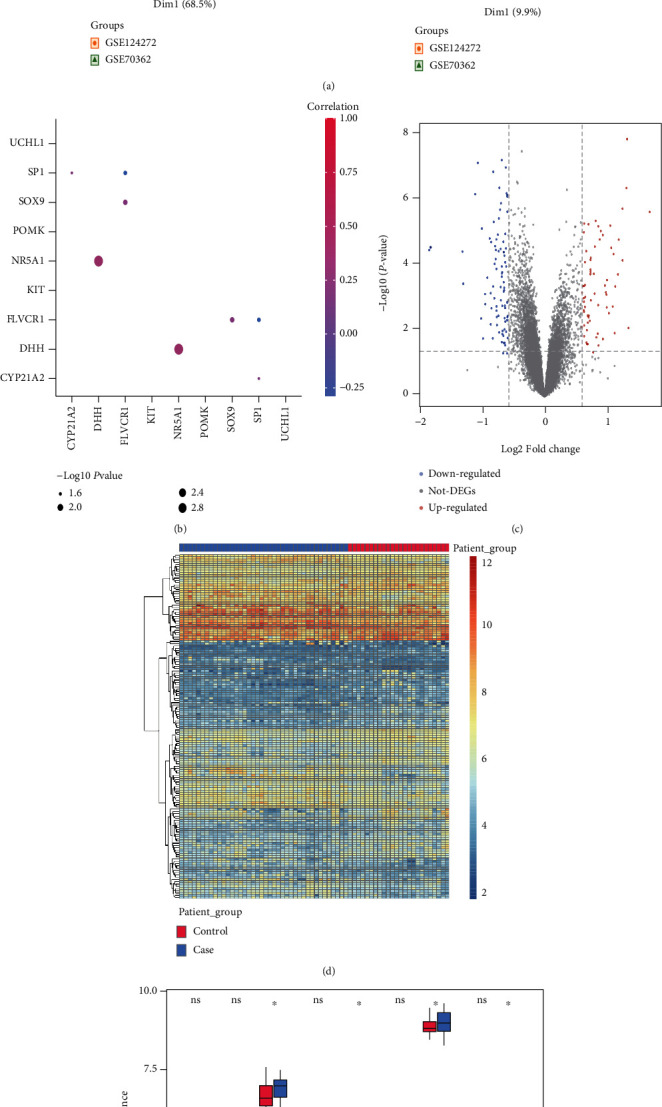
Genes related to mitochondrial dysfunction. (a): box plot and PCA of gene expressions before and after batch effect removal in the GEO data. (b): correlations among mitochondrial dysfunction genes. Colors indicate correlations. A pinker color means stronger correlation. Node size indicates -log10 (*P*-value), and a larger node means higher significance. (c): volcano plots of DEGs, *x*-axis: log2FoldChange, *y*-axis: -log10 (adjust P-value); red, gray and blue nodes indicate the differentially expressed genes are upregulated, insignificant, and downregulated, respectively. (d): heat maps of DEGs, blue: degeneration group, red: control group. (e): histograms of expressions of mitochondrial dysfunction genes in test group and control group; *x*-axis: mitochondrial dysfunction genes, *y*-axis: gene expression level; red: test group, blue: control group. P <0.05: significant level. (f): PPI network of mitochondrial dysfunction genes.

This study procedure was conducted methodically based on the steps outlined in the flow diagram (Figure 1). To build a panorama of mitochondrial dysfunction genes in all samples, we first integrated the expression profiles from two data sets. Given the severe batch effect of data sets from different sources, we first corrected the batch effect of original data, and log-normalized the data. Results showed that the expression distributions of all samples after the above processing were distributed in consistent ways, which helped improve the accuracy and robustness of downstream analyses (Figure 2(a)). The integrated data set after the removal of batch effect contained 40 IDD samples and 24 control samples.

### 3.2. Panorama of Mitochondrial Dysfunction Genes

The expression levels of mitochondrial dysfunction genes were sent to correlation analysis. Results showed gene NR5A1 and gene DHH were very highly positively correlated, and gene SOX9 and gene FLVCR1, and gene CYP21A2 and gen SP1 were highly correlated (Figure 2(b)).

To analyze the differences between the control and test groups, we obtained 152 DEGs, including 67 upregulated genes and 85 downregulated genes (Figure 2(c)). A heat map of these DEGs was plotted, and the two groups of samples were well differentiated using gene clustering (Figure 2(d)). Histograms of mitochondrial dysfunction genes between the test group and the control group were plotted. Results showed FLVCR1, NR5A1, SOX9, and UCHL1 were significantly different (Figure 2(e)). A PPI network with 9 mitochondrial dysfunction genes from the gene expression profile was plotted, of which 7 genes were interactive (Figure 2(f)).

### 3.3. Construction of Risk Model

**Figure 3:**
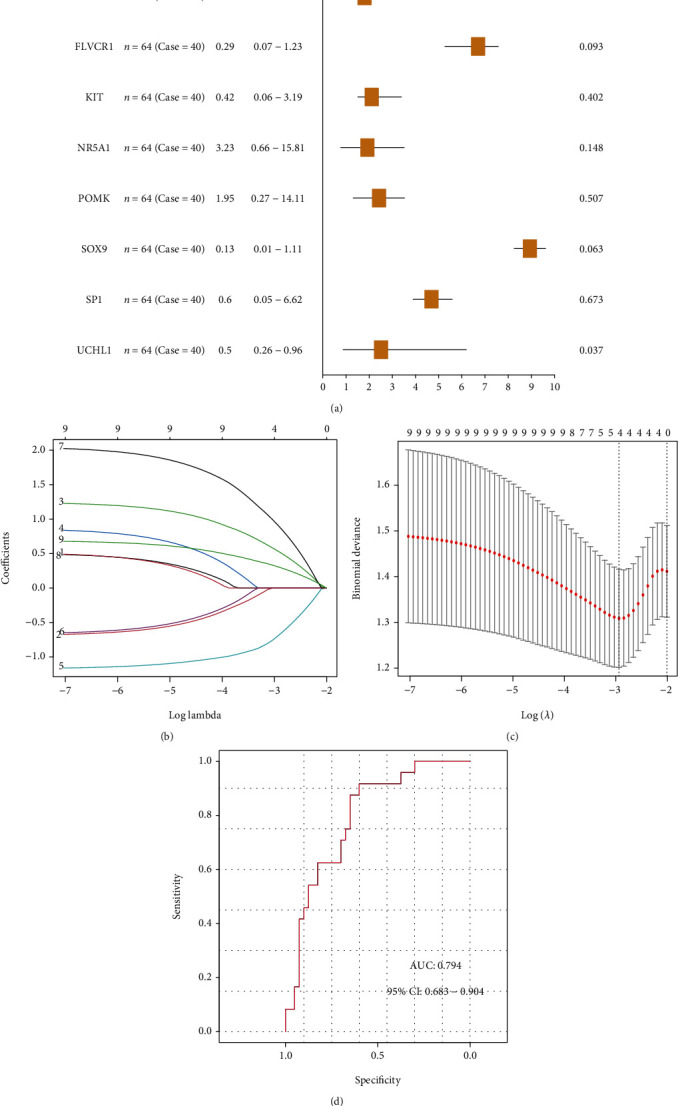
Construction of IDD model. (a): single-factor regression analysis. (b)–(c): mitochondrial dysfunction genes identified by LASSO regression. (d): ROC curve of IDD diagnostic model.

To analyze the effects of mitochondrial dysfunction genes on IDD patients, we conducted logistic single-factor regression analysis, and identified 9 mitochondrial dysfunction genes that largely affected IDD, including 4 significant genes (FLVCR1, NR5A1, SOX9, UCHL1) (Figure 3(a)). The coefficients of the 9 genes were calculated using LASSO (Figures 3(b)–3(c)). The gene expression level of each gene was multiplied by the corresponding coefficient, and then added together, forming a IDD predicted score. The final predicted score of each sample was calculated, and plotted on an ROC curve. Results showed the prediction curve call well predict IDD (Figure 3(d)).

### 3.4. Biological Differences between Groups

**Figure 4:**
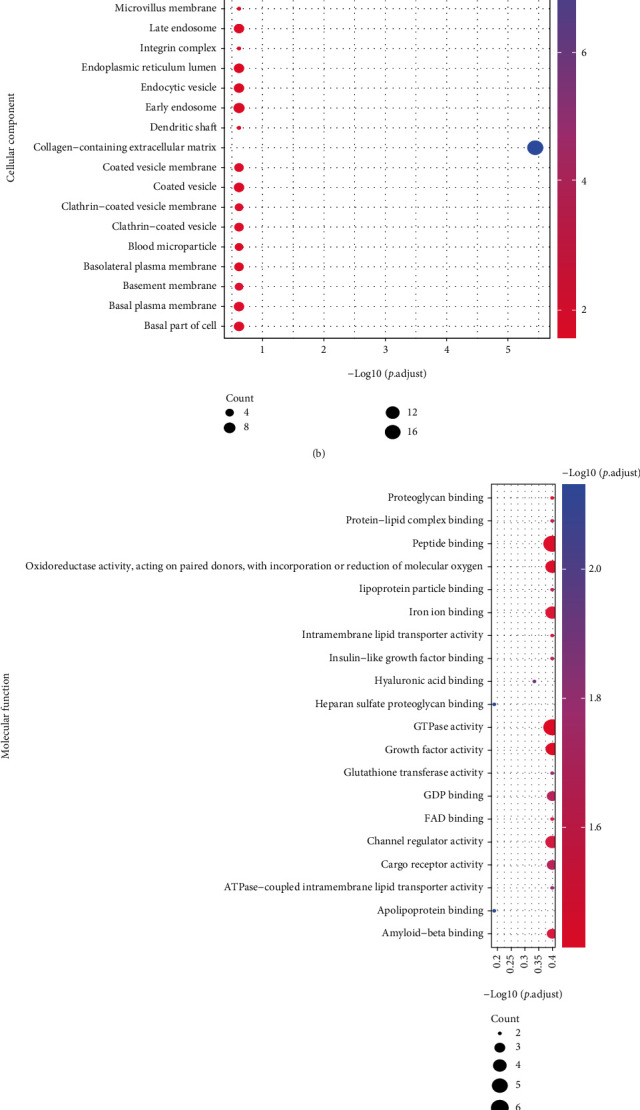
GO and KEGG enrichment analysis. (a): BP enrichment results, *x*-axis: -log10 (*p* value), *y*-axis: GO terms, node colors indicate -log10 (p value), node size indicates the number of genes contained the current GO Term. (b): CC enrichment results. (c): MF enrichment results. (d): KEGG enrichment results.

To explore the effects of between-group DEGs on the biological functions of patients, we first annotated the GO functions of DEGs (Table S1). Results showed these DEGs are mainly enriched in bioprocesses of extracellular matrix organization, epithelial cell migration, endodermal cell differentiation, defense response to symbiont (Figure 4(a)), cellular components of collagen-containing extracellular matrix (Figure 4(b)), and molecular functions of collagen binding, glycosaminoglycan binding, extracellular matrix structural constituent, heparin binding (Figure 4(c)). These DEGs were also enriched in KEGG pathways related to Focal adhesion, PI3K-Akt signaling pathway, Chemical carcinogenesis - receptor activation, and ECM-receptor interaction (Figure 4(d), Table S2).

**Figure 5:**
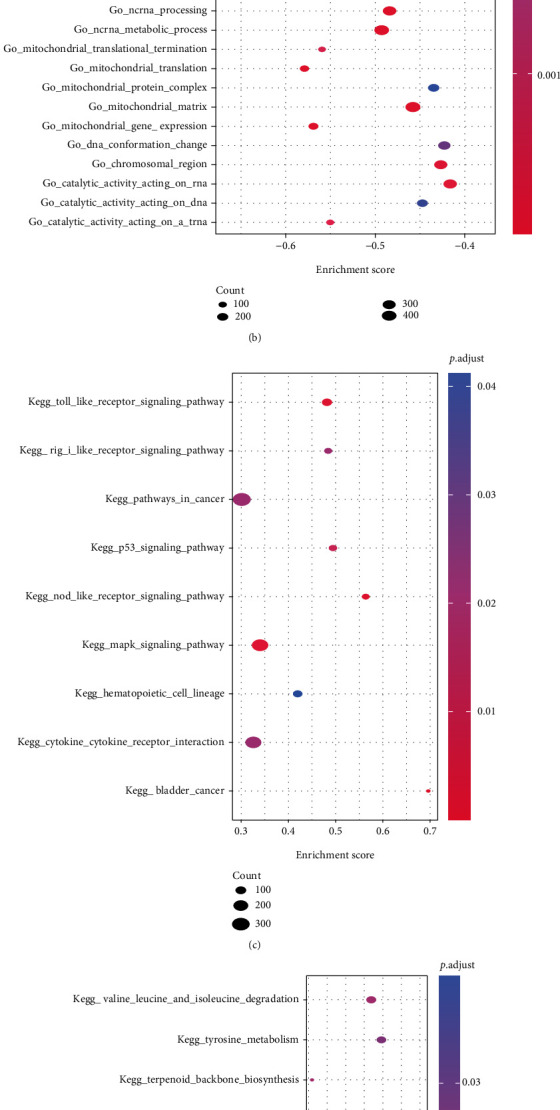
GSEA and GSVA. (a)–(b): GSEA-GO, *x*-axis: gene ratio, *y*-axis: GO terms, colors indicate -log10 (p value), node size indicate the number of genes enriched in GO terms. GO pathways activated (a) and inhibited (b) in the tested group. (c)–(d): GSEA-KEGG, *x*-axis: gene ratio, *y*-axis: KEGG pathway, node size indicates the number of genes enriched in the pathway, node colors indicate -log10 (p value). KEGG pathways activated (c) and inhibited (d) in the tested group. (e)–(f): heat map of functional scores in GSVA, *x*-axis: samples, *y*-axis: biological functions, node colors indicate the corresponding function is activated (red) or inhibited (blue). Red: control group, blue: test group.

Then all genes between groups were subjected to GSEA (Table S3). Results showed the following biological processes were significantly enriched between groups. In the IDD samples, bioprocesses including response to oxidative stress, response to virus, acute inflammatory response, wound healing, and positive regulation of protein kinase activity were activated, while bioprocesses including tRNA metabolic process, RNA modification, ribosome biogenesis, and ribonucleoprotein complex biogenesis were inhibited (Figures 5(a)–5(b)). Moreover, MAPK signaling pathway, toll like receptor signaling pathway, and nod like receptor signaling pathway were activated, but purine metabolism, DNA replication, and ECM receptor interaction pathways were inhibited (Figures 5(c)–5(d)).

The results of GSVA were basically consistent with the GSEA results. Especially, cellular response to hydroperoxide, copper ion import, positive regulation of platelet activation, and ipaf inflammasome complex were activated in the disease samples, while cysteine type exopeptidase activity, and oxidative DNA demethylation were inhibited (Figure 5(e)). In the meantime, olfactory transduction, nod like receptor signaling pathway, and gap junction were activated, but pyruvade metabolism, riboflavin metabolism, and DNA replication were inhibited (Figure 5(f)).

### 3.5. WGCNA and PPI Network

**Figure 6:**
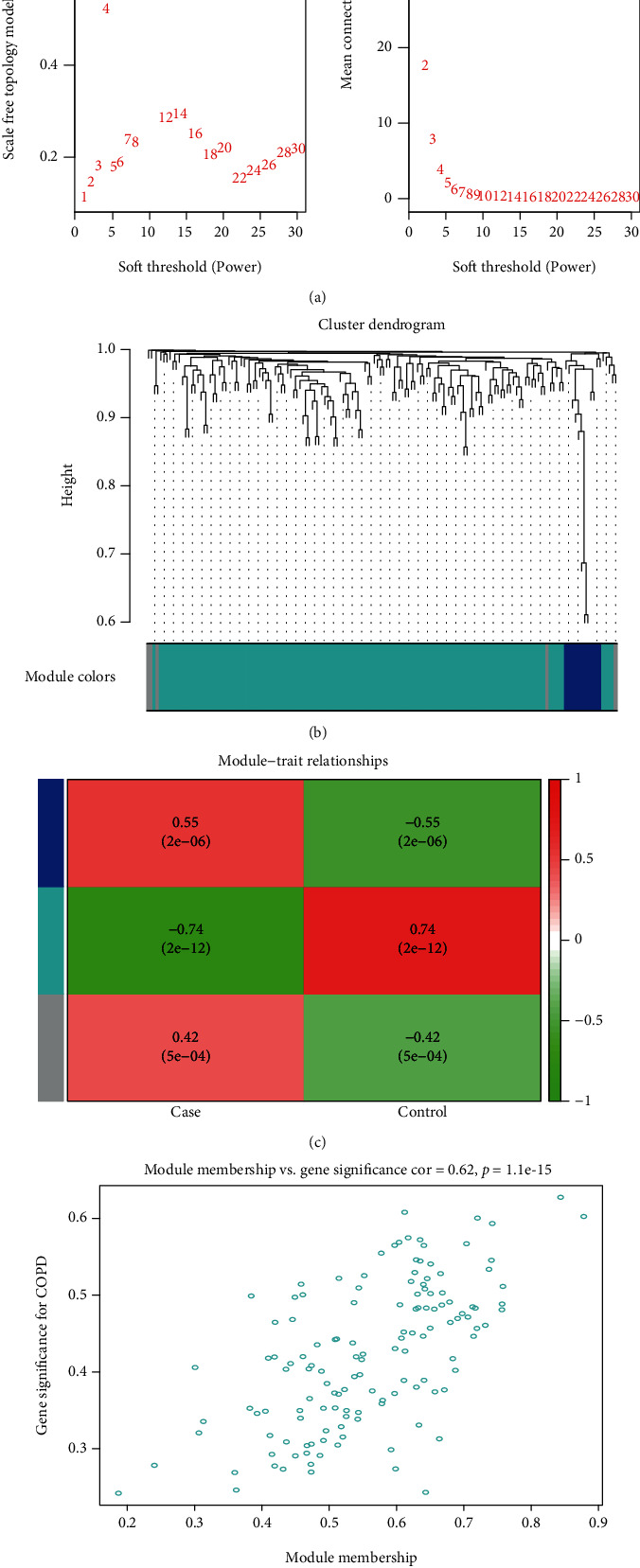
WGCNA. (a): WGCNA of threshold screening. (b): coexpression gene clustering. (c): correlations between gene clusters and diseased patients; (d): correlation analysis between most significant gene clusters and IDD.

To probe into the associations between DEGs, we first subjected the DEGs to WGCNA (Figure 6(a)). One coexpression gene module was identified (Figure 6(b)), from which the gene set with the highest correlation was picked out and used in subsequent analysis (Figures 6(d)–6(d)), which returned 136 key genes.

**Figure 7:**
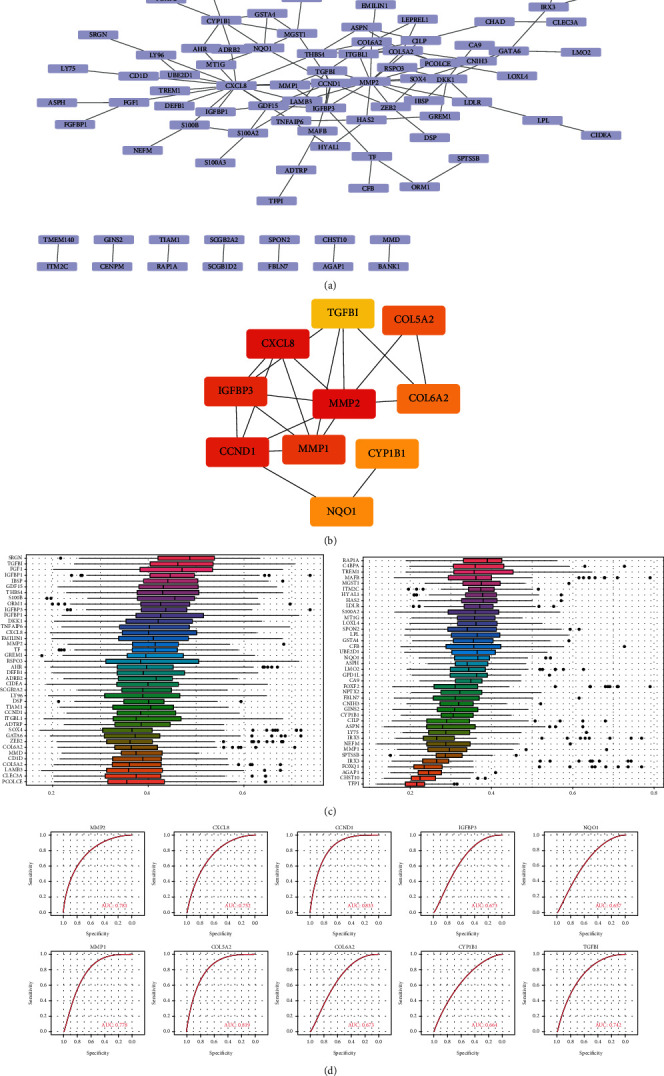
PPI network. (a): PPI network of key genes; (b): subnetwork of PPI network. (c): analysis of similarities of gene functions in subnetwork of PPI network; *x*-axis: magnitude of correlations; *y*-axis: gene name. (d): ROC curve of hub genes.

From the PPI network involving the 136 key genes, we analyzed the effects of the genes on all steps of the life process, including biosignal transfer, gene expression regulation, energy and substance metabolism, and cell cycle regulation. Through visualization on Cytoscape (Figure 7(a)), the network contains 120 interaction pairs and 87 genes. Specifically, CCND1 is closely connected with 11 DEGs, and CXCL8 is closely associated with 13 DEGs. The function interactive subnetworks were extracted on CytoHubba (Figure 7(b)), which included 10 genes. The functional similarities among the genes on the PPI network were analyzed (Figure 7(c)). Results showed the genes in PPI were highly associated in terms of functions. An ROC curve with the 10 hub genes was plotted, which showed these genes can well differentiate the two groups of samples (Figure 7(d)).

### 3.6. Analysis of Immune Infiltration

**Figure 8:**
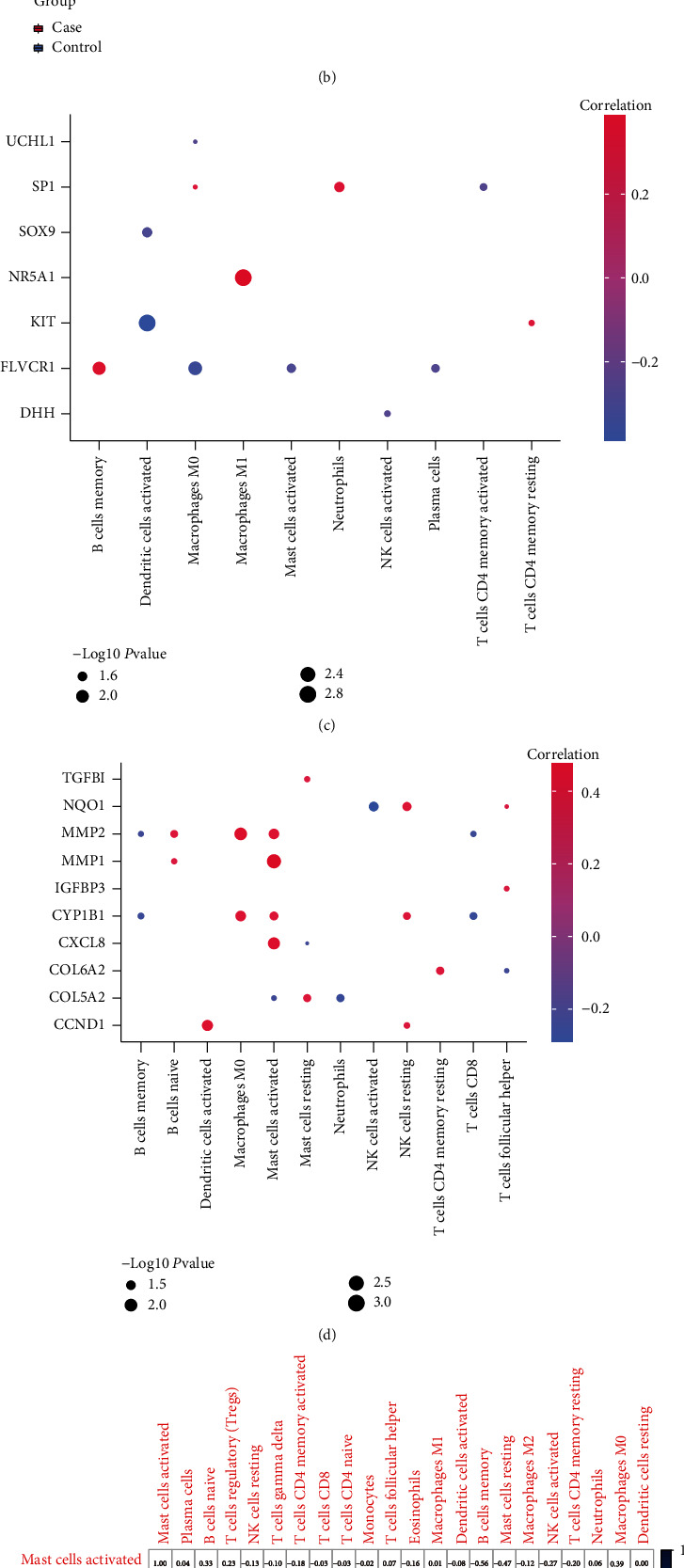
Immune infiltration analysis. (a): Accumulative immune cell concentrations in the test group and control group. Colors indicate immune cells. *x*-axis: id of patients. (b): histograms of immune cell concentrations; *x*-axis: immune cells; *y*-axis: cell concentration; red: IDD samples; blue: control samples. (c): correlations between mitochondrial dysfunction genes and immune cells; *x*-axis: immune cells, *y*-axis: mitochondrial dysfunction genes. Node colors indicate the magnitude of correlation, node sizes indicate the significance level. (d): correlations between hub genes and immune cells; *x*-axis: immune cells, *y*-axis: hub genes. Node colors indicate the magnitude of correlation, node sizes indicate the significance level. (e)–(f): correlations of immune cell concentrations in the test group (e) and control group (f); red: negative correlation, blue: positive correlation.

CIBESORT (Figure 8(a)) showed the concentrations of mast cells activated were significantly the highest in IDD patients. Compared with the control group, the level of T cells CD4 memory resting was the lowest in the patients (Figure 8(b)). Furthermore, the correlations of mitochondrial dysfunction genes or hub genes with immune cell contents were calculated. Results showed macrophages M0, and dendritic cells activated were both closely associated with the expressions of several mitochondrial dysfunction genes (Figure 8(c)). Macrophages M0, NK cells resting, and mast cells activated were positively correlated with multiple hug genes, and B cells memory, and T cells CD8 were negatively correlated with several hub genes (Figure 8(d)). Moreover, the correlations of immune cell contents between the control group and the tested group were computed. Results showed the Mast cells activated contents in the patients were significantly correlated with several types of immune cells (Figure 8(e)). In the control group, the content of T cells CD4 naive was significantly correlated with several other types of immune cells (Figure 8(f)).

### 3.7. Two Mitochondrial Dysfunction Modes Identified Using Mitochondrial Dysfunction Genes

**Figure 9:**
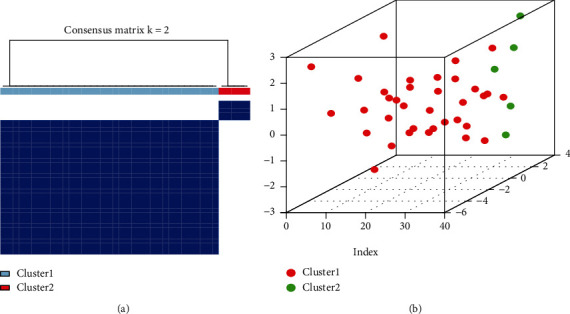
Consistency clustering of IDD patients according to mitochondrial dysfunction. (a): consistency clustering, blue: cluster1, red: cluster2. (b): PCA of cluster1 and cluster2, red: cluster1, blue: cluster2.

With ConsensusClusterPlus from the R software and based on the 9 mitochondrial dysfunction genes, the mitochondrial dysfunction modes were identified using consistency clustering, and two modes were identified (cluster1, cluster2)(Figure 9(a)). Cluster1 contains 35 samples, and cluster2 involves 5 samples. PCA showed the two clusters were significantly different (Figure 9(b)).

**Figure 10:**
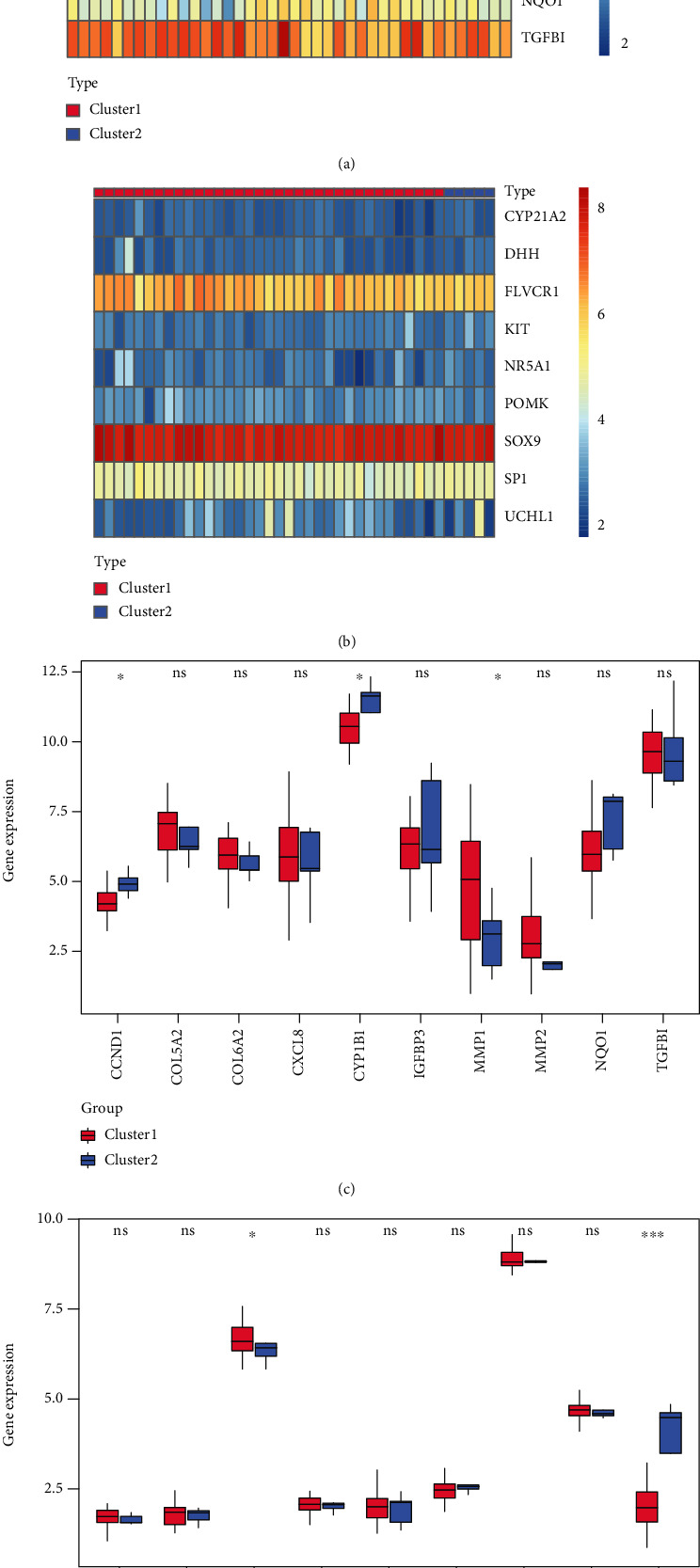
Differences of hub genes between two groups of IDD patients. (a)/(c): heatmaps and histograms of hub-related gene expressions in cluster1 and cluster2; (c): *x*-axis: hub genes; *y*-axis: gene expression level; red: cluster1, blue: cluster2. (b)/(d): heatmaps and histograms of mitochondrial dysfunction gene expressions in cluster1 and cluster2; (d): *x*-axis: mitochondria dysfunction genes; *y*-axis: gene expression level; red: cluster1, blue: cluster2. P <0.05: significance.

The differences of hub genes and mitochondrial dysfunction genes between the two clusters were compared. Results showed CCND1 and CYP1B1 were significantly down-expressed in cluster1, and MMP1 was significantly up-expressed in cluster2 (Figures 10(a) and 10(c)). In the meantime, UCHL1 expression of cluster1 was significantly lower than that of cluster2, and FLVCR1 expression of cluster1 was higher than that of cluster2 (Figures 10(b) and 10(d)).

### 3.8. Validation of the Mitochondria Dysfunction Genes

**Figure 11:**
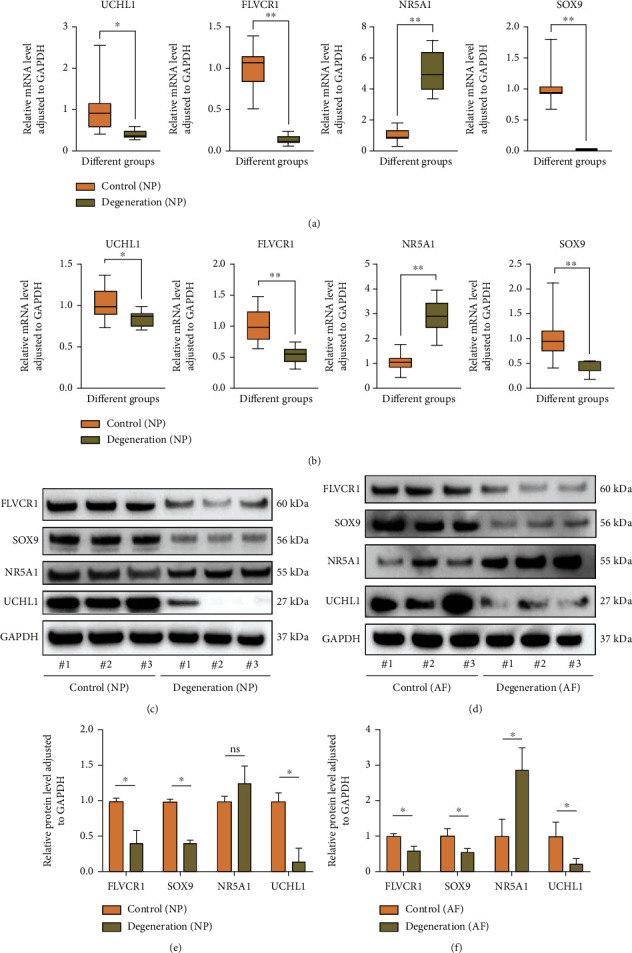
Validation of differential genes of mitochondrial dysfunction between normal intervertebral disc tissues and IDD tissues. (a)/(c): qPCR and WB results of normal intervertebral disc tissues and IDD nucleus pulposus tissues. (b)/(d): qPCR and WB results of normal intervertebral disc tissues and IDD anulus fibrous tissues. (e)–(f): quantitative analysis of protein expression (relative to GAPDH)∗p<0.05; ∗∗p <0.01.

To validate the identified mitochondria dysfunction genes, we collected 36 human intervertebral disc tissues RNA including 12 from patients with degenerated discs of Pfirrmann level I or II and 24 from patients with degenerated discs of Pfirrmann level III to V. QPCR showed either in nucleus pulposus tissues or in anulus fibrous tissues, the mRNA levels of NR5A1 were upregulated in both degenerative NP tissues and AF tissues than in control groups. While the mRNA levels of FLVCR1, SOX9 and UCHL1 were downregulated. These results are shown in Figures 11(a)–11(b). Additionally, the protein level validation results were consistent with the gene level results. Western blot showed the relative expression levels of FLVCR1, SOX9 and UCHL1 proteins in both NP cells and AF cells of the degeneration group were significantly lower than that in the control group. But that of NR5A1 was higher than in the control group in fibrous anulus cells while there was no significant difference between these two groups in pulposus cells (Figures 11(c)–11(f)).

## 4. Discussion

Intervertebral disc (IVD) is a complex fibrocartilaginous tissue that connects adjacent vertebral bodies and maintains mechanical loading to enable spinal motion. In a healthy IVD, the balance between anabolic and catabolic processes maintains ECM homeostasis. However, aging and persistent mechanical stress can disrupt IVD metabolism, forcing an imbalance between the expressions of catabolic factors (e.g. pro-inflammatory cytokines and matrix metalloproteinases) and anabolic mediators (e.g. growth factors), leading to the loss of ECM homeostasis, destruction of macromolecules, and the subsequent development of IDD [38]. IDD plays an important role in spine-related diseases, and worsens with age. More than 80% of IDDs exhibit degeneration-related changes in people over 50 years [39].

The widely-used treatment strategies for IDD include physiotherapy, drug therapy and surgery. However, these approaches have limited effects to reverse the IDD progression with current treatments. Therefore, it is necessary to fully understand the etiology of IDD. The important bioprocesses are related to immune response, innate immune response, cell division, mitochondrial homeostasis and cell proliferation. More studies have proved that mitochondrial dysfunction is pivotal in accelerating IDD. Mitochondria, a double-layer organelle in eukaryotic cells, is mainly involved in tricarboxylic acid cycle (TCA) and provides the body with adenosine-triphosphate enzyme (ATP). ATP is the most important energy source of cells, but mitochondria, while providing ATP, also produce ROS that cause oxidized stress response [40]. Along with cell aging, mitochondrial DNA injuries will induce mitochondrial dysfunction, destroying the redox balance, so ROS aggravate cell injuries, forming a vicious circle ‘cell aging -- mitochondrial dysfunction -- ROS maladjustment accelerated cell aging'. In contrast, mitochondrial respiratory uncoupling reduces the production of H_2_O_2_, thereby delaying the replicative senescence of cells. Reportedly, the expression levels of mitochondrial function genes (including substrate dehydrogenase, cytochrome and substrate vectors) are significantly changed in aged intervertebral disc tissues.

The function of mitochondria depends on their morphological structure, and abnormal changes in the morphological structure can lead to mitochondrial dysfunction. The dynamic balance between mitochondrial fusion and division is one major link in maintaining cell homeostasis, and the destruction of this balance can cause a series of diseases [41, 42]. Mitochondrial homeostasis requires mitofusion and optic atrophy-associated protein 1 (OPA1) regulation [42, 43]. When mitochondria divide, the outer mitochondrial membrane molecule Fis1 recruits dynamin-related protein 1 (DRP1), a mitochondrial division regulator, translocating it to the outer mitochondrial membrane and enriching it at the site of division [43, 44]. OPA1 and mitochondrial fusion occur when disc tissue is subjected to excessive mechanical loading. Down-regulation of proteins leads to mitochondrial fusion disorders, resulting in NP cell damage [44]. When intervertebral disc cells are under hypoxia, DRP1 migrates from the cytoplasm to mitochondria, aggravating mitochondrial dysfunction [8]. Therefore, an intervention targeting DRP1 is one of the feasible strategies to prevent IDD. Inhibition of DRP1 by siRNA or mitochondrial division inhibitor 1 (Mdivi-1) can prevent programmed necrosis in NP cells or AF cells exposed to excessive pressure [45, 46].

ROS are involved in signal transduction and metabolism of intervertebral disc cells to regulate death and senescence. Under physiological conditions, the production and clearance of intracellular ROS are under dynamic balance [47]. When the balance is broken, ROS levels in cells will exceed physiological thresholds, subjecting the cells to a state of oxidative stress dysfunction and excessive mitochondrial ROS level in intervertebral discs [48–53]. Mitochondrial DNA (mtDNA) and oxidative damage of respiratory enzymes lead to mitochondrial dysfunction, forming a vicious cycle [54]. In intervertebral disc tissues, ROS overproduction can lead to loss of proteoglycan collagen and accelerate IDD progression [55]. H_2_O_2_ significantly increases lysosomal membrane permeability in rat NP cells, leading to ROS overproduction and apoptosis [56]. Excessive increase in ROS in the intervertebral disk can cause oxidative damage to disk cells, leading to activation of NLRP3 inflammatory vesicles and increased interleukin (IL)1 release, exacerbating the inflammatory response. In the IL-1*β* inflammatory environment, ROS expression is elevated in NP cells, and ROS activates p53/P21 and P16/*κ*b pathways to accelerate intervertebral disc cell senescence [57].

The emergence of bioinformatics methods has accelerated the research progress on the mechanisms of human diseases. In our study, comparative analyses of mitochondria dysfunction DEGs between normal individuals and IDD patients were performed. We integrated two databases GSE7036 and GSE124272, which contained tissue and blood sequencing data. The analysis of immune infiltration showed that compared with the control group, the concentrations of mast cells activated were significantly the highest in IDD patients. Previous studies have shown that mast cells might play a key role in the repair of the injured anulus fibrosus and subsequent disc degeneration [58]. GSEA analysis indicated that the biological process of response to oxidative stress is activated in degeneration samples. In addition, GSVA analysis obtained a similar result: cellular response to hydroperoxide was overactivated in patient samples. These results further confirmed the role of oxidative stress response caused by mitochondrial dysfunction in the progression of IDD. Further analysis showed that 152 DEGs were obtained between degeneration group and control group, including 67 upregulated genes and 85 downregulated genes. Four mitochondrial dysfunction genes (FLVCR1, SOX9, UCHL1, NR5A1) were identified and validated at the gene and protein levels. After an extensive literature review of these 4 genes, we found no report about the associations of three genes with IDD (SOX9 was proved to be associated with IDD). Hence, our findings may be a new clue for the diagnosis and treatment of IDD.

Reportedly, mitochondrial injuries during cartilage degeneration will induce abnormal expressions in SOX9 and its downstream genes. We found in either IDD tissues or peripheral blood, the SOX9 gene expressions were significantly different between the degeneration group and the control group, and the SOX9 expression gradually decreased with the aggravation of degeneration. SOX9 was first noted in skeletogenic mesenchymal progenitors for its role in fate determination and differentiation within the chondrocyte lineage [59]. SOX9 efficiently binds to single or double HMG-box site(s) in DNA and thereby transactivates its target genes such as Col2a1 and aggrecan (Acan), which have stage-specific features for intervertebral disc cartilage development [60, 61].

Inactivation of Sox9 during early stages of chondrogenesis in Col2Cre-expressing chondrocytes showed that cells cannot express SOX9 targets, including Col2a1 and Acan [62]. Observations in the notochord during axial skeletogenesis complement the findings in chondrocytic cells. Sox9 deletion prevents matrix-rich notochordal sheath formation and results in notochordal cell death [63]. It is suggested that SOX9 regulates cell survival and differentiation in the inner AF during disc development [64]. These studies affirmed that SOX9 is critical in cartilage formation and vertebral column development [65]. Moreover, Sox9 deletion in Acan-expressing cells of adult mice results in proteoglycan loss, disc compression, and downregulation of various ECM-related genes [66]. A recent study from Maria demonstrated that SOX9 mutant mice experience early-onset, progressive disc degeneration characterized by increased cell death, alterations in ECM organization, and distinct transcriptomic changes in the NP and AF compartments.

Feline leukemia virus subgroup C receptor-1 (FLVCR1), a member of the SLC49 family of 4 paralogous genes, is a cell surface heme exporter essential for erythropoiesis and systemic iron homeostasis. It encodes two heme exporters: FLVCR1a [67], which localizes to the plasma membrane, and FLVCR1b, which localizes to mitochondria [68]. Upon the occurrence of mitochondrial dysfunction, FLVCR1b abnormality will lead to the accumulation of free heme, high levels of Hb and heme can be used to mark neovasculogenesis in herniated nucleus pulposus, which further promotes nucleus pulposus degeneration through heme iron-dependent cell death. This finding is consistent with our validation results at the gene and protein levels. When IDD occurs, the FLVCR1 expression is low in both NP cells and AF cells.

NR5A1, also known as steroidogenic factor-1 (SF-1) or adrenal 4-binding protein (Ad4BP), was initially identified as a steroidogenic cell-specific transcription factor that regulates the transcription of steroidogenic genes. Recent research proves NR5A1 can regulate nearly all glycolytic genes to adjust the nutrition metabolism of tissues. When normal IDD tissues are under a low oxygen condition, the physiologically hypoxic intervertebral disc rely on the hypoxia-inducible factor (HIF) family of transcription factors to mediate cellular responses to changes in oxygen tension. Mechanistically, HIF1 is the master regulator of glycolytic metabolism and cytosolic lactate levels. In addition, HIF1 regulates mitochondrial metabolism by promoting flux through the tricarboxylic acid cycle, inhibiting downsteam oxidative phosphorylation and controlling mitochondrial health through modulation of the mitophagic pathway [69]. The destruction to the low-oxygen condition of IDD will damage tricarboxylic acid cycle, so the energy metabolism is destroyed in cells. NR5A1, one of the important genes of glycolysis regulation and control, is significantly upregulated to correct energy metabolic disorders and maintain the normal energy metabolism in the intervertebral disc. Our bioinformatic analysis also validated this view that NR5A1 expression is significantly higher in IDD tissues.

Ubiquitin C-terminal hydrolase L1 (UCHL1) is a deubiquitinating enzyme that was originally found in neurons. UCHL1 was first studied in Alzheimer's disease (AD). UCHL1 protein expression decreased in the cerebral cortex of AD patients [70]. Our study confirmed that UCHL1 expression in IDD tissues decreases, which may be related to the regulating role of UCHL1 in mitochondria. Previous studies show that when UCHL1 is specifically knocked out, the key proteins involved in mitochondrial oxidative phosphorylation are significantly reduced, suggesting that UCHL1 may be involved in regulation of mitochondrial content and function [71]. When dUCH (a homolog of human UCHL1) was specifically knocked down in motor neurons, the motor neuron cells exhibited aberrant morphology and function of mitochondria, such as mtDNA depletion, an increase in mitochondrial size, and overexpression of antioxidant enzymes [72].

There were several limitations of our study. Firstly, only few RNA-sequencing datasets of IDD are available on the GEO database. Clinical data such as disease phenotype and radiological data are unavailable from published studies and public databases. Secondly, all of these analyses were obtained by data mining based on a series of bioinformatic algorithms, and we did not provide the original sequencing data with the clinical samples, so we cannot evaluate the quality of the sequencing samples, such as the actual degrees of IDD. Thirdly, the exact molecular mechanisms of candidate mitochondrial dysfunction related genes in IDD need to be further investigated using in-depth in vitro and in vivo scientific experiments. Finally, the nature of retrospective research limits the clinical value of this work, indicating multicentre or prospective studies are imperative to elucidate the relationship between mitochondrial dysfunction and IDD.

## 5. Conclusions

In this study, we used bioinformatics methods to compare tissue and peripheral blood RNA-seq data between IDD and control groups. We have successfully constructed a risk model which can call well predict IDD and have elaborated on the different groups of immune infiltrates and identified two modes of mitochondrial dysfunction. In addition, we identified four genes, verified the reliability by molecular biology experiment, associated with mitochondrial dysfunction. These findings may provide a new perspective for the diagnosis and treatment of IDD.

## Figures and Tables

**Table 1:** Primers used for RT-qPCR.

FLVCR1	F:5′- GGAACTTGAATCCAGCCAGAGAA -3′
	R:5′- GTCCGTTGTATCCATAAGGTAGCA -3′

NR5A1	F:5′- GACAGGGAGAAGTTGAGCAGGTAT -3′
	R:5′- TTGGGTGGGAGAGGGAATCAGT -3′

UCHL1	F:5′- GCTCAAGCCGATGGAGATCAAC -3′
	R:5′- ACTGCGTGAATAAGTCCGATTGTG -3′

SOX9	F:5′- GAGCAGCGAAATCAACGAGAAACT -3′
	R:5′- ACAAAGTCCAAACAGGCAGAGAGA -3′

GAPDH	F:5′- ACTTTGGTATCGTGGAAGGACTCA -3′
	R:5′- CCAGTAGAGGCAGGGATGATGTT -3′

## Data Availability

Publicly available datasets were analyzed in this study. This data can be found here: https://www.ncbi.nlm.nih.gov/geo/query/acc.cgi?acc=GSE70362;https://www.ncbi.nlm.nih.gov/geo/query/acc.cgi?acc=GSE124272.

## Supplementary Materials

Supplementary Materials Table S1 GO terms enrichment analysis. Table S2 KEGG pathway enrichment analysis. Table S3 GSEA.
