# Supplementary material for: Integrative Bioinformatics Analysis Revealed Mitochondrial Dysfunction-Related Genes Underlying Intervertebral Disc Degeneration
Source: Oxid Med Cell Longev. 2022 Oct 11;2022:1372483. doi: 10.1155/2022/1372483 (PMC9578809; doi:10.1155/2022/1372483)
Supplement: Supplementary Materials — Table S1 GO terms enrichment analysis. Table S2 KEGG pathway enrichment analysis. Table S3 GSEA. [file 1372483.f1.zip › Supplementary material Table S1.docx]

**Table S1. GO terms enrichment analysis**

| **Category** | **ID** | **Description** | **p** |
| --- | --- | --- | --- |
| BP | GO:0030198 | extracellular matrix organization | 2.87E-07 |
| BP | GO:0043062 | extracellular structure organization | 2.96E-07 |
| BP | GO:0045229 | External encapsulating structure organization | 3.16E-07 |
| BP | GO:0010631 | epithelial cell migration | 5.28E-07 |
| BP | GO:0090132 | epithelium migration | 5.84E-07 |
| BP | GO:0090130 | tissue migration | 6.89E-07 |
| BP | GO:0001667 | ameboidal-type cell migration | 1.38E-05 |
| BP | GO:0035987 | endodermal cell differentiation | 1.93E-05 |
| BP | GO:0007492 | endoderm development | 2.32E-05 |
| BP | GO:0051607 | defense response to virus | 2.81E-05 |
| BP | GO:0140546 | defense response to symbiont | 2.81E-05 |
| BP | GO:0001706 | endoderm formation | 4.84E-05 |
| BP | GO:0030199 | collagen fibril organization | 5.30E-05 |
| BP | GO:0060394 | negative regulation of pathway-restricted SMAD protein phosphorylation | 8.74E-05 |
| BP | GO:0061430 | bone trabecula morphogenesis | 8.74E-05 |
| BP | GO:0009615 | response to virus | 8.80E-05 |
| BP | GO:0031589 | cell-substrate adhesion | 8.80E-05 |
| BP | GO:0055094 | response to lipoprotein particle | 0.000106325 |
| BP | GO:0071402 | cellular response to lipoprotein particle stimulus | 0.000119801 |
| BP | GO:0070141 | response to UV-A | 0.000143089 |
| BP | GO:0030282 | bone mineralization | 0.000213696 |
| BP | GO:0110148 | biomineralization | 0.000228124 |
| BP | GO:0001503 | ossification | 0.000231171 |
| BP | GO:0051701 | biological process involved in interaction with host | 0.000257371 |
| BP | GO:0090136 | epithelial cell-cell adhesion | 0.000262918 |
| BP | GO:0006805 | xenobiotic metabolic process | 0.000295957 |
| BP | GO:0052126 | movement in host environment | 0.000364763 |
| BP | GO:0071466 | cellular response to xenobiotic stimulus | 0.000368571 |
| BP | GO:0009410 | response to xenobiotic stimulus | 0.00047334 |
| BP | GO:0035455 | response to interferon-alpha | 0.000503017 |
| BP | GO:0090101 | negative regulation of transmembrane receptor protein serine/threonine kinase signaling pathway | 0.000533748 |
| BP | GO:0060343 | trabecula formation | 0.00057924 |
| BP | GO:0071404 | cellular response to low-density lipoprotein particle stimulus | 0.00057924 |
| CC | GO:0062023 | collagen-containing extracellular matrix | 1.55E-08 |
| MF | GO:0005201 | extracellular matrix structural constituent | 5.15E-05 |
| MF | GO:0005518 | collagen binding | 0.000176865 |
| MF | GO:0008201 | heparin binding | 0.000279056 |
| MF | GO:0005539 | glycosaminoglycan binding | 6.85E-05 |
