# Supplementary material for: Integrative Bioinformatics Analysis Revealed Mitochondrial Dysfunction-Related Genes Underlying Intervertebral Disc Degeneration
Source: Oxid Med Cell Longev. 2022 Oct 11;2022:1372483. doi: 10.1155/2022/1372483 (PMC9578809; doi:10.1155/2022/1372483)
Supplement: Supplementary Materials — Table S1 GO terms enrichment analysis. Table S2 KEGG pathway enrichment analysis. Table S3 GSEA. [file 1372483.f1.zip › Supplementary material Table S2.docx]

**Table S2. KEGG pathway enrichment analysis**

| **Category** | **ID** | **Description** | **p** |
| --- | --- | --- | --- |
| KEGG_PATHWAY | hsa04510 | Focal adhesion | 0.00012197 |
| KEGG_PATHWAY | hsa05219 | Bladder cancer | 0.000610723 |
| KEGG_PATHWAY | hsa04512 | ECM-receptor interaction | 0.001516222 |
| KEGG_PATHWAY | hsa01524 | Platinum drug resistance | 0.005206463 |
| KEGG_PATHWAY | hsa04115 | p53 signaling pathway | 0.005206463 |
| KEGG_PATHWAY | hsa05133 | Pertussis | 0.006005757 |
| KEGG_PATHWAY | hsa02010 | ABC transporters | 0.009072876 |
| KEGG_PATHWAY | hsa04979 | Cholesterol metabolism | 0.012110644 |
| KEGG_PATHWAY | hsa05207 | Chemical carcinogenesis - receptor activation | 0.015977431 |
| KEGG_PATHWAY | hsa05417 | Lipid and atherosclerosis | 0.017013149 |
| KEGG_PATHWAY | hsa00480 | Glutathione metabolism | 0.018053167 |
| KEGG_PATHWAY | hsa04978 | Mineral absorption | 0.01975068 |
| KEGG_PATHWAY | hsa04151 | PI3K-Akt signaling pathway | 0.019875724 |
| KEGG_PATHWAY | hsa05145 | Toxoplasmosis | 0.022457624 |
| KEGG_PATHWAY | hsa05204 | Chemical carcinogenesis - DNA adducts | 0.028447508 |
| KEGG_PATHWAY | hsa00982 | Drug metabolism - cytochrome P450 | 0.031730516 |
| KEGG_PATHWAY | hsa04215 | Apoptosis - multiple species | 0.037562912 |
| KEGG_PATHWAY | hsa00980 | Metabolism of xenobiotics by cytochrome P450 | 0.038866575 |
| KEGG_PATHWAY | hsa05165 | Human papillomavirus infection | 0.039242122 |
| KEGG_PATHWAY | hsa05418 | Fluid shear stress and atherosclerosis | 0.044442501 |
| KEGG_PATHWAY | hsa04610 | Complement and coagulation cascades | 0.048135778 |
