# Supplementary material for: Integrative Bioinformatics Analysis Revealed Mitochondrial Dysfunction-Related Genes Underlying Intervertebral Disc Degeneration
Source: Oxid Med Cell Longev. 2022 Oct 11;2022:1372483. doi: 10.1155/2022/1372483 (PMC9578809; doi:10.1155/2022/1372483)
Supplement: Supplementary Materials — Table S1 GO terms enrichment analysis. Table S2 KEGG pathway enrichment analysis. Table S3 GSEA. [file 1372483.f1.zip › Supplementary material Table S3.docx]

**Table S3. GSEA**

| **ID** | **ES** | **NES** | **p** |
| --- | --- | --- | --- |
| go_mitochondrial_matrix | -0.457884279 | -1.904110156 | 1.00E-10 |
| go_ncrna_metabolic_process | -0.492781408 | -2.045475373 | 1.00E-10 |
| go_ncrna_processing | -0.483958084 | -1.981850634 | 1.00E-10 |
| go_ribonucleoprotein_complex_biogenesis | -0.464929379 | -1.922662987 | 1.00E-10 |
| go_mitochondrial_gene_expression | -0.569044763 | -2.150777824 | 1.11E-10 |
| go_mitochondrial_translation | -0.579045715 | -2.154554158 | 5.28E-10 |
| go_ribosome_biogenesis | -0.485278005 | -1.952517599 | 2.15E-09 |
| go_leukocyte_migration | 0.371830797 | 1.780834997 | 2.34E-09 |
| go_rrna_metabolic_process | -0.515207736 | -2.01145098 | 2.42E-09 |
| go_response_to_virus | 0.416054044 | 1.952088118 | 3.51E-09 |
| kegg_bladder_cancer | 0.696271675 | 2.391667999 | 3.13E-07 |
| kegg_nod_like_receptor_signaling_pathway | 0.563773776 | 2.081606993 | 1.35E-05 |
| kegg_toll_like_receptor_signaling_pathway | 0.481687501 | 1.917681718 | 4.02E-05 |
| kegg_dna_replication | -0.650553359 | -1.953302016 | 6.39E-05 |
| kegg_mapk_signaling_pathway | 0.339997181 | 1.546527636 | 9.23E-05 |
| kegg_purine_metabolism | -0.445447368 | -1.664732395 | 0.000165302 |
| kegg_p53_signaling_pathway | 0.494304171 | 1.848734789 | 0.000518641 |
| kegg_rig_i_like_receptor_signaling_pathway | 0.484187225 | 1.785429335 | 0.000977466 |
| kegg_cytokine_cytokine_receptor_interaction | 0.325790472 | 1.476821219 | 0.001269058 |
| kegg_nucleotide_excision_repair | -0.563740181 | -1.717970717 | 0.001297103 |
